# Does MMP-9 Gene Polymorphism Play a Role in Pituitary Adenoma Development?

**DOI:** 10.1155/2017/5839528

**Published:** 2017-01-17

**Authors:** Brigita Glebauskiene, Rasa Liutkeviciene, Alvita Vilkeviciute, Loresa Kriauciuniene, Silvija Jakstiene, Egle Zlatkute, Abdonas Tamosiunas, Reda Zemaitiene, Paulina Vaitkiene, Dalia Zaliuniene

**Affiliations:** ^1^Medical Academy, Department of Ophthalmology, Lithuanian University of Health Sciences, Kaunas, Lithuania; ^2^Medical Academy, Neuroscience Institute, Lithuanian University of Health Sciences, Kaunas, Lithuania; ^3^Medical Academy, Radiology Department, Lithuanian University of Health Sciences, Kaunas, Lithuania; ^4^Medical Academy, Lithuanian University of Health Sciences, Kaunas, Lithuania; ^5^Medical Academy, Cardiology Institute, Lithuanian University of Health Sciences, Kaunas, Lithuania

## Abstract

*Purpose*. To determine if the MMP-9 genotype has an influence on development of pituitary adenoma (PA).* Methodology*. The study enrolled *n* = 86 patients with PA and *n* = 526 healthy controls (reference group). The genotyping of MMP-9 was carried out using the real-time polymerase chain reaction method.* Results*. Our data demonstrated that the MMP-9 (–1562) C/C genotype was more frequent in PA group than in healthy controls (81.4% versus 64.6%, *p* = 0.002); C/C genotype was more frequently present in PA females compared to healthy control females, 81.5% versus 64.6%, *p* = 0.018, as well. MMP-9 (–1562) C/C genotype was frequently observed for all subgroups: noninvasive and invasive, nonrecurrence, and inactive PA compared to healthy controls: 81.8% versus 64.6%, *p* = 0.021; 81.0% versus 64.6%, *p* = 0.041; 81.8% versus 64.6%, *p* = 0.005; 100.0% versus 64.6%, *p* < 0.001, respectively. MMP-9 (–1562) C/C genotype was more frequent in inactive PA compared to active PA: 100.0% versus 71.4%; *p* < 0.001.* Conclusion*. MMP-9 (–1562) C/C genotype plays a role in nonrecurrence, inactive, and invasive as well as in nonivasive PA development.

## 1. Introduction

Most of the pituitary tumours are pituitary adenomas (PAs): benign, slow-growing neoplasms that arise from cells of the pituitary gland. Pituitary adenomas have the high incidence, following the gliomas and meningiomas. In recent years, the frequency of PA has been increasing, particularly in younger age groups [1, 2]. PA accounts for 15 to 20% of primary brain tumours. PA may grow large and extend into the surrounding structures resulting in neurological complications including visual impairment [3]. In addition, pituitary adenomas may be distinguished anatomically as intrapituitary, intrasellar, diffuse, and invasive [4]. Invasive adenomas which account for approximately 35% of all pituitary neoplasms may invade the dura mater, cranial bone, or sphenoid sinus [5]. PA is a disease of multifactorial etiology, the occurrence of which is influenced by genetic factors, hormonal stimulation, growth factors, and so forth. Recently, great attention in the PA pathogenesis has been drawn to the search for new epigenetic and genetic factors [6]. To invade, tumour cells must undergo several changes in molecular pathways in accordance with invasion-associated cellular activities, namely, cell-cell adhesion, cell-matrix adhesion and ectopic survival, migration, and proteolysis [7]. Invasive factors involve the heparinase, serine proteinases, cathepsins, and matrix metalloproteinases* (MMPs)* [8].* MMPs* are capable of degrading most of the components of the extracellular matrix which may play an important role in the extracellular matrix remodeling during angiogenesis. A particular interest has been focused on* MMP-9* due to its ability to degrade components of the basement membrane components, such as type IV collagen. There are 24 different genes defined that code the expression of proteases of* MMP* family [9]. The expression in transcription level depends on gene promoter mutations and various transcription factors. A transition of C to T at the 1562-bp position upstream of the transcription initiation site (–1562 C/T) of* MMP-9* (NCBI SNP identification number rs3918242) has shown having an effect on promoter activity. Transition of C nucleotide to T nucleotide causes more difficulties for nucleic protein complex binding to DNA strain in the presence of T allele. It was determined that once C allele mutates to T allele, a promoter activity increases 1.5 times [10].

Few studies have analyzed MMP-9 expression in PA [11–13] and in other tumours such as prostate and gastric cancer [14–16]. MMP-9 expression was reported to be significantly higher in invasive pituitary adenomas [17–20]. Also, some studies have been carried out to look for the possible association between the MMP-9 (–1562 C/T) gene polymorphism and prostate [14], breast [21], gastric [22–24], colorectal [25, 26], and lung cancer [27]. However, results of the published studies are controversial. To our knowledge, there have been no studies that investigated the association between the MMP-9 (–1562 C/T) gene polymorphism and PA development. Therefore, the aim of this study was to determine the association between the MMP-9 (–1562 C/T) gene polymorphism and the development of PA.

## 2. Materials and Methods

Permission (number P2-9/2003) to undertake the study was obtained from the Kaunas Regional Biomedical Research Ethics Committee. The study was conducted in the Departments of Ophthalmology and Neurosurgery, the Hospital of Lithuanian University of Health Sciences Kaunas Clinics.

The study comprised 86 subjects with a diagnosis of pituitary adenoma and 526 healthy subjects in the control group.

The control group was designed by taking into consideration the distribution of age and gender in the pituitary adenoma group. Therefore, the medians of the patients' age of the control group and the pituitary group did not differ statistically significantly (*p* > 0.05).

Demographic data of the study subjects are presented in Table 1.

The inclusion criteria of the PA group were as follows: (1) determined and confirmed PA via MRI; (2) a patient's general good condition; (3) a patient's consent to take part in the study; (4) age ≥ 18 years, (5) no other brain or other localization tumours.

### 2.1. Invasiveness Evaluation

The analysis of all pituitary adenomas was based on MR imaging findings. The suprasellar extension and sphenoid sinus invasion by PAs were classified according to the Wilson-Hardy classification (the Hardy classification, modified by Wilson) [28]. The degree of suprasellar and parasellar extension was graded as stages A–E. The degree of sellar floor erosion was graded as grades I–IV. Grade III, localized sellar destruction, and grade IV, diffuse destruction, were considered as invasive PA. The Knosp classification system was used to quantify invasion of the cavernous sinus, in which only grades 3 and 4 define true invasion of the tumour into the cavernous sinus. In grade 0, there is no cavernous sinus involvement; in grades 1 and 2, the tumour pushes into the medial wall of the cavernous sinus but does not go beyond a hypothetical line extending between the centres of the two segments of the internal carotid artery (grade 1) or it goes beyond such a line, but without passing a line tangent to the lateral margins of the artery itself (grade 2); in grade 3, the tumour extends laterally to the internal carotid artery within the cavernous sinus; in grade 4, there is total encasement of the intracavernous carotid artery [29]. Thus grade III and IV tumours were considered to be invasive.

### 2.2. Activeness and Recurrence Evaluation

The analysis of all pituitary adenomas was based on histopathological findings of PA and hormone levels in the blood serum before surgery. All 100 subjects were categorized into two groups, active or inactive PA. Active PA group was not distributed to smaller groups by increase of specific hormone because dominant tumours were prolactinomas and others would not fill the optimal space in our study. Since some of the 100 subjects had already had surgery in recent years, we categorized them by recurrence of pituitary adenoma into two groups, with PA and without recurrence.

### 2.3. Control Group Formation

The age- and gender-matched control group comprised 526 subjects randomly selected from the following projects:

1. The international Health, Alcohol and Psychosocial Factors in Eastern Europe (HAPPIE) project involving the Kaunas population aged 45–74 years run by the Laboratory of Population Research, Institute of Cardiology, LUHS.
2. The international Countrywide Integrated Noncommunicable Disease Intervention (CINDI) project involving the Lithuanian population aged 25–65 years run by the Laboratory of Preventive Medicine, Institute for Biomedical Research, LUHS.
3. The Kaunas Healthy Ageing Study involving the Kaunas population older than 65 years run by the Clinic of Geriatrics and the Laboratory of Molecular Cardiology, Institute of Cardiology, LUHS.

### 2.4. DNA Extraction and Genotyping

The DNA extraction and analysis of the gene polymorphism of* MMP-9* were carried out at the Laboratory of Molecular Cardiology at the Institute of Cardiology of the LUHS for control group and at the Laboratory of Ophthalmology at the Institute of Neuroscience of the LUHS for the PA patient group. DNA was extracted from 200 *μ*L venous blood (white blood cells) using a DNA purification kit based on the magnetic beads method (MagJET Genomic DNA Kit, Thermo Scientific) or the silica-based membrane technology utilizing a genomic DNA extraction kit (GeneJET Genomic DNA Purification Kit, Thermo Scientific), according to the manufacturer's recommendations.

The genotyping of* MMP-9* (–1562 C/T) was carried out using the real-time polymerase chain reaction (PCR) method. For the genotyping of* MMP-9* (–1562 C/T) (Rs3918242), the following were used: 2x TaqMan® Universal Master Mix, nuclease-free water, and the following primers and fluorescently labeled allele specific probes: forward primer 5′-CAGATCACTTGAGTCAGAA-3′, reverse primer 5′-GGTGTAGTATCACTCTGTCA-3′, probe “C” allele 5′ FAM-TGGCGCACGCCTATAATACCA-MGB 3′, and probe “T” allele 5′ VIC-TGGCGCATGCCTATAATACCAGC-MGB 3′ (Applied Biosystems, Warrington, UK). PCR conditions included predenaturation at 95°C for 10 minutes followed by 40 cycles of 95°C for 30 seconds, 58°C for 30 seconds, and 72°C for 30 seconds. Genotyping was performed using the HT 7900 real-time PCR quantification system (Applied Biosystems, USA).

To ensure an internal control, 20 samples were sequenced at the Sequencing Center of the Institute of Biotechnology.

### 2.5. RNA Extraction and MMP-9 mRNA Expression Analysis

Twenty-eight PA samples were surgically resected in the Department of Neurosurgery, Hospital of Lithuanian University of Health Sciences Kaunas Clinics (Lithuania), and were confirmed histologically. PA tissue samples were snap-frozen in liquid nitrogen prior to RNA extraction. Total RNA was purified using TRIzol Reagent (Ambion, Life Technologies). cDNA synthesis was performed using total RNA (2 *μ*g) and random hexamer primers (ThermoFisher Scientific) with the RevertAid H Minus M-MuLV Reverse Transcriptase (ThermoFisher Scientific) in a final volume of 40 *μ*L, according to the manufacturer's recommendations. For inhibition of mRNA degradation RiboLock RNase inhibitor (ThermoFisher Scientific) was used. MMP-9 mRNA expression was analyzed using quantitative real-time RT-PCR TaqMan probe assay (assay number Hs00234579_m1) in 3 replicates on 7500 Fast Real-Time PCR detection system (Applied Biosystems). One endogenous control (GAPDH TaqMan probe assay number Hs0 2758991_g1) was used. MMP-9 expression levels in PA were determined by the comparative Ct method (2^−ΔΔCt^). The expression of MMP-9 was normalized to GAPDH and calibrated using reference sample (“FirstChoice Human Brain Reference RNA” (Ambion)).

### 2.6. Statistical Analysis

Statistical analysis was performed using the SPSS/W 20.0 software (Statistical Package for the Social Sciences for Windows, Inc., Chicago, Illinois, USA). The data are presented as absolute numbers with percentages in brackets and as medians with min. and max. values. The frequencies of genotypes (in percentage) are presented in Table 2.

Hardy-Weinberg analysis was performed to compare the observed and expected frequencies of* MMP-9* using the *χ*^2^ test in all groups. The distributions of* MMP-9* SNPs in the PA and control groups were compared using the *χ*^2^ test or the Fisher exact test. Binomial logistic regression analysis was performed to estimate the impact of genotypes on PA development. Odds ratios and 95% confidence intervals are presented. The selection of the best genetic model was based on the Akaike Information Criterion (AIC); therefore, the best genetic models were those with the lowest AIC values. Kruskal–Wallis test was used to reveal the difference across medians of MMP-9 mRNA expression in all hormone groups. Differences were considered statistically significant when *p* < 0.05.

## 3. Results

The genotyping of MMP-9 (–1562) C/T was performed in patients with PA and in the control group subjects (Table 2). The distribution of the analyzed MMP genotypes and allele frequencies in the control group but not in PA group matched the Hardy-Weinberg equilibrium. MMP-9 (–1562) C/T gene polymorphism analysis in the overall group revealed differences in the genotype distribution between patients with PA and control group patients (*p* = 0.003). The genotype C/C was more frequent in PA group than in healthy controls (81.4% versus 64.6%, *p* = 0.002) and the genotype C/T was less frequent in PA group compared to healthy control group (14.0% versus 32.1%, *p* < 0.001) (Table 2).

MMP-9 (–1562) C/T gene polymorphism analysis between females and males with PA did not reveal any statistically significant differences in the genotypes (C/C, C/T, and T/T) distribution (as follows: 81.5%, 14.8%, and 3.7% versus 81.2%, 12.5%, and 6.2%) (Table 3). A comparison of MMP-9 genotype distribution in healthy females and females with PA revealed significant differences. MMP-9 (–1562) C/C genotype was more frequently present in PA females compared to healthy control females: 81.5% versus 64.6%; *p* = 0.018; and C/T was less frequent in PA females compared to healthy females: 14.8% versus 32.3%; *p* = 0.01. MMP-9 (–1562) T/T genotype did not show any statistically significant differences when healthy females and females with PA were compared. When analyzing genotypes distribution in men, only MMP-9 (–1562) C/T genotype distribution showed statistically significant difference among men with PA and healthy men: 12.5% versus 31.9%; *p* = 0.023.

Binomial logistic regression analysis in the patients with PA and in the control group was performed (Table 4). This analysis revealed that there were statistically significant variables in the codominant (*p* = 0.01), dominant (*p* = 0.003), overdominant (*p* = 0.001), and additive (*p* = 0.018) models of the patients with PA and in the control group.

Binomial logistic regression analysis in the patients with PA and in the control group by gender was also performed (Table 5). There were statistically significant variables in the codominant (*p* = 0.037) and overdominant (*p* = 0.032) models of males. In females this analysis revealed that the codominant (*p* = 0.012), dominant (*p* = 0.017), overdominant (*p* = 0.012), and additive (*p* = 0.044) variables were statistically significant.

MMP-9 (–1562) C/C genotype was frequently observed in noninvasive, nonrecurrence, and inactive PA subgroups compared to healthy controls: 81.8% versus 64.6%; *p* = 0.021; 81.0% versus 64.6%; *p* = 0.041; 81.8% versus 64.6%; *p* = 0.005; 100.0% versus 64.6%; *p* < 0.001, respectively. On the other hand, there were no differences between noninvasive and invasive and nonrecurrence and recurrence PA subgroups except when compared with inactive and active PA. There was statistically significant difference between patients with inactive PA and active PA. MMP-9 (–1562) C/C genotype was more frequent in inactive PA compared to active PA: 100.0% versus 71.4%; *p* < 0.001 (Tables 6, 7, and 8).

Further analysis revealed that MMP-9 (–1562) C/T genotype was less frequent in invasive, nonrecurrence, recurrence, and inactive PA subgroups compared to healthy controls: 9.5% versus 32.1%; *p* = 0.001; 15.2% versus 32.1%; *p* = 0.004; 10.0% versus 32.1%; *p* = 0.047; 0% versus 32.1%; *p* < 0.001, respectively (Tables 6, 7, and 8).

The analysis of PA subgroups showed only one statistically significant difference between inactive and active PA. MMP-9 (–1562) C/T genotype was more frequent in active PA subgroup compared to inactive one: 21.4% versus 0%; *p* = 0.007 (Table 8).

Binomial logistic regression analysis in noninvasive PA and in the control group was performed (Table  9). In noninvasive PA group this analysis revealed that the codominant (*p* = 0.045), dominant (*p* = 0.025), overdominant, and additive (*p* = 0.028) variables were statistically significant. Binomial logistic regression analysis in the patients with invasive PA and in the control group was performed as well (Table  9, shown in Supplementary Data available online at https://doi.org/10.1155/2017/5839528). In invasive PA group this analysis showed that the codominant (*p* = 0.007), dominant (*p* = 0.036), and overdominant (*p* = 0.005) variables were statistically significant.

Binomial logistic regression analysis in inactive PA and in the control group was performed as well as in active PA and in the control group (Table  10, shown in Supplementary Data). There was no statistical significance of variables in analysis of MMP-9 (–1562) in both groups by activity of PA.

Binomial logistic regression analysis performed in nonrecurrence PA and in the control group showed that the codominant (*p* = 0.006), dominant (*p* = 0.007), overdominant (*p* = 0.006), and additive (*p* = 0.016) variables were statistically significant (Table  11, shown in Supplementary Data). There was no statistical significance of variables in analysis of MMP-9 (–1562) in PA with recurrence.

**Figure 1:**
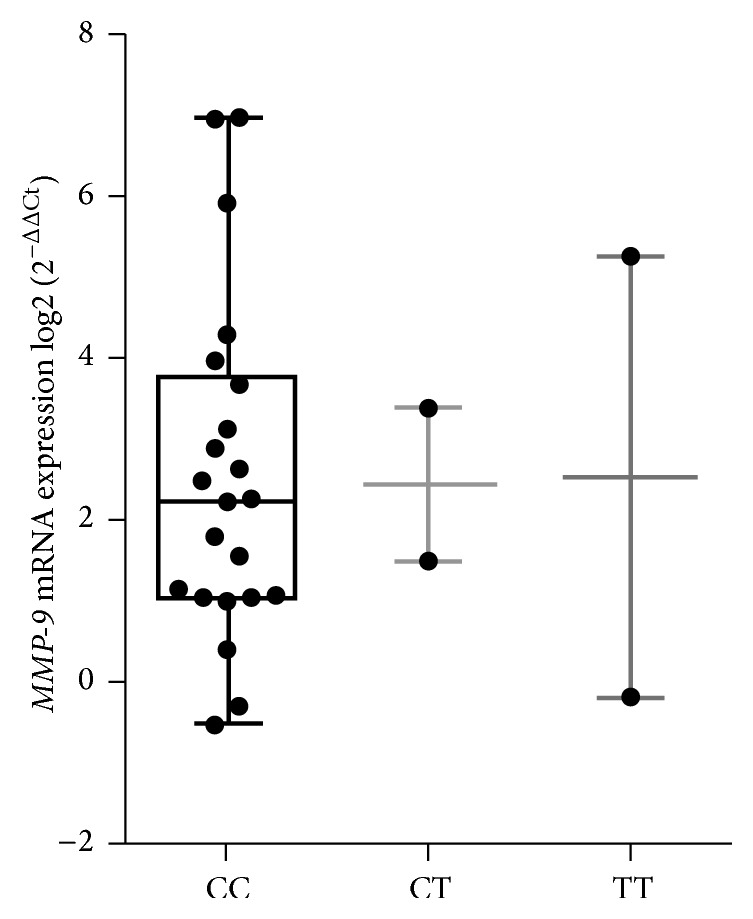
MMP-9 mRNA expression in pituitary adenoma (PA).

MMP-9 mRNA expression analysis in pituitary adenomas was performed. Therefore, the results of MMP-9 mRNA expression did not reveal any statistically significant differences between three genotypes of MMP-9 (C/T) polymorphism (C/C, C/T, and T/T). Lower expression was noticed in patients with MMP-9 C/C genotype (36.4% (8/22)), but differences between groups were not statistically significant (*p* = 0.9) (Figure 1).

*MMP-9* expression in different groups of genotypes (CC, CT, and TT) of MMP-9 gene in patients with PA: the horizontal bars represent the mean values; the spots represent the amount of samples.

## 4. Discussion 

The impact of MMP-9 (–1562) C/T gene polymorphism on the development of various tumours was analyzed in few studies [14, 21–27]. In addition,* MMP-9* expression has been shown to be significantly higher in invasive PAs compared to noninvasive ones [17–20]. In our previous study we analyzed 84 PA patients and 318 age- and sex-matched controls for the –1306 C/T polymorphism in the* MMP-2 *promoter. Our results demonstrated that* MMP-2 (*–*1306) C/T *genotype was more frequently present in PA females compared to healthy control females: 33.66% versus 49.1%; *p* = 0.041 [30].

On the basis of these findings, we sought to examine whether the polymorphism in the* MMP-9 (*–*1562) *promoter could have an impact on the risk of PA development.

Our data demonstrated that the MMP-9 (–1562) C/C genotype, which causes lower gene expression, was more frequent in PA group than in healthy controls (81.4% versus 64.6%, *p* = 0.002); C/C genotype was more frequently present in PA females compared to healthy control females: 81.5% versus 64.6%; *p* = 0.018, as well. It is very interesting that MMP-9 (–1562) C/C genotype was frequently observed for all subgroups: noninvasive and invasive, nonrecurrence, and inactive PA compared to healthy controls: 81.8% versus 64.6%; *p* = 0.021; 81.0% versus 64.6%; *p* = 0.041; 81.8% versus 64.6%; *p* = 0.005; 100.0% versus 64.6%; *p* < 0.001, respectively. MMP-9 (–1562) C/C genotype was more frequent in inactive PA compared to active PA: 100.0% versus 71.4%; *p* < 0.001. Therefore, we may hypothesize that MMP-9 gene polymorphism plays a role in noninvasive/invasive, nonrecurrence, and inactive PA development.

To our knowledge, no studies have been carried out analyzing the impact of MMP-9 (–1562) C/T gene polymorphism on the development of PA. Previous studies on the morphogenesis of PA have drawn attention to the role of MMP-9 expression but not to the MMP-9 C/T gene polymorphism in the development of PA, especially considering PA invasiveness.

Kawamoto et al. have observed the incidence of tumour cells secreting MMP-9 to be significantly higher in invasive pituitary adenomas than in noninvasive ones [17]. Gong et al. [20] have analyzed 73 pituitary tumour specimens and have found MMP-9 mRNA expression to be significantly increased in the majority of invasive pituitary adenomas. Liu et al. have found MMP-9 score of invasive case (4.1 ± 0.4) to be significantly higher than those (2.6 ± 0.2; *p* < 0.01) without invasion [18]. Hussaini et al. have demonstrated an increase in the expression level and activity of MMP-9 in invasive nonfunctioning PAs and HP75 cell line [19]. Turner et al. also have reported MMP-9 expression to be higher in invasive macroprolactinomas (*p* = 0.003) when compared with noninvasive macroprolactinomas or normal anterior pituitary gland [31]. Significantly higher MMP-9 expression was detected in invasive prolactinomas (*p* = 0.004) by Gültekin et al. [32]. Some authors have not found an association of MMP-9 expression and tumour invasiveness [33]. Few studies have also been carried out to look for an association between the MMP-9 –1562 C/T polymorphism and the risk of other human tumours. Schveigert et al. in their research have found MMP-9 –1562 polymorphism CC variant to be associated with prostate cancer tumour differentiation grade [14]. Zheng et al. have reported CT and TT genotypes of the MMP-9 gene (C–1562T) as high-risk genotypes for solid breast tumour invasion and metastasis progression [15]. In another study Matsumura et al. have found that the presence of T allele at MMP-9 –1562 site was significantly associated with tumour progression and invasive phenotype of gastric cancer among Japanese population [23]. In another study Kubben et al. did not find any significant association between MMP-9 –1562 C/T polymorphism and gastric cancer risk among Caucasian population [24]. Jafari et al. in their study concluded that T allele may be the risk factor for lung cancer progression [27]. Xing et al. have reported that the MMP-9–1562C>T polymorphism affects lymph node metastasis of colorectal cancer [26]. On the basis of these findings, we hypothesized that the –1562 C/T polymorphism in MMP-9 might also have impact on individual susceptibility to PA.

It is the first study to examine MMP-9 (–1562 C/T) polymorphism in patients with PA. Numerous genetic studies have been carried out to assess an association between the MMP-9 expression and PA clinical features, but results remain controversial [17–20, 31, 32]. Additionally, we analyzed MMP-9 expression using mRNA determination in resected PA of 28 patients and explored the correlation between MMP-9 gene mRNA expression and different genotypes of MMP-9 (–1562 C/T) polymorphism. In our study we found that MMP-9 (–1562) C/C genotype was more frequently observed in PA group than in healthy controls with a tendency for lower mRNA activity in most of PA samples with CC genotype. Unfortunately, these findings did not show any statistical significance (*p* = 0.9). Zhang and colleagues determined that once C allele mutates to T allele a promoter activity increases [10], and in such way CC genotype may cause lower promoter activity resulting in lower MMP-9 expression. These findings correlate with our study results which show higher frequency of CC genotype in inactive, noninvasive, and nonrecurrence PA groups suggesting that CC genotype could be associated with decreased MMP-9 expression, as well. However, there are more MMP-9 gene polymorphisms which may interact and influence MMP-9 expression, so further research with the bigger sample size is needed to analyze other MMP-9 gene polymorphisms as risk factors for PA development.

To the best of our knowledge, this is the first study that examined the relationship between the MMP-9 (–1562) C/T gene polymorphism and development of PA. For the first time, we have found that MMP-9 (–1562) C/C genotype was frequently observed for all subgroups: noninvasive and invasive, nonrecurrence, and inactive PA compared to healthy controls. Therefore, MMP-9 (–1562) C/T gene polymorphism might play an important role in the development on PA but further research is required to be repeated in the bigger sample size.

## Supplementary Material

Binomial logistic regression analysis in noninvasive/invasive, inactive/active and nonrecurrence/recurrence PAs.

## Figures and Tables

**Table 1:** Demographic characteristics of patients with pituitary adenoma (PA) and the control group subjects.

Characteristics	Patients	Controls	*p*
Sample size	86	526	
Age (year) (min./max. median)	19/87/52.5	25/87/51	0.88
Gender			
Females, *n* (%)	54 (62.8)	316 (60.1)	0.633
Males, *n* (%)	32 (37.2)	210 (39.9)	
Invasiveness			
Invasive, *n* (%)	42 (48.8)		
Noninvasive, *n* (%)	44 (51.2)		
Recurrence			
Recurrence, *n* (%)	20 (23.3)		
Nonrecurrence, *n* (%)	66 (76.7)		
Activity			
Active, *n* (%)	56 (65.1)		
Inactive, *n* (%)	30 (34.9)		

**Table 2:** Frequency of MMP-9 (–1562 C/T) genotype in the patients with pituitary adenoma (PA) and in the control group.

Gene marker	Genotype/allele	Frequency (%)				
		Control group *n* (%) (*n* = 526)	*p* HWE	PA group *n* (%) (*n* = 86)	*p* HWE	*p* value
*MMP-9 (*–*1562) Rs3918242*	Genotype					
	C/C	340(64.6)^*∗*^	0.469	70(81.4)^*∗*^	**0.0029**	*X* ^2^ = 11.788 *p* = **0.003**
	C/T	169(32.1)^*∗∗*^		12(14.0)^*∗∗*^		
	T/T	17 (3.2)		4 (4.7)		
	Total	526 (100)		86 (100)		
	Allele					
	C	849 (80.70)		152 (88.37)		
	T	203 (19.30)		20 (11.63)		

MMP, matrix metalloproteinase; *p* value, significance level (alpha = 0.05); *p* value HWE, significance level (alpha = 0.05) by Hardy-Weinberg equilibrium.

^*∗*^ *p* = 0.002.

^*∗∗*^ *p* < 0.001.

**Table 3:** Frequency of MMP-9 (–1562 C/T) genotype in the patients with pituitary adenoma (PA) and in the control group by gender.

Gene marker	Genotype/allele	Frequency (%)							
		Control group *n* (%)		*p* HWE	*p* value	PA group *n* (%)		*p* HWE	*p* value
		Females *N* = 316	Males *N* = 210			Females *N* = 54	Males *N* = 32		
*MMP-9 (*–*1562) Rs3918242*	Genotype								
	C/C	**204 (64.6)** ^1^	136 (64.8)	0.991	0.868	**44 (81.5)** ^1^	26 (81.2)	0.836	1.00
	C/T	**10 (32.3)** ^2^	**67(31.9)** ^3^		0.928	**8(14.8)** ^2^	**4 (12.5)** ^3^		1.00
	T/T	10 (3.2)	7 (3.3)		0.915	2 (3.7)	2 (6.2)		0.626
	Total	316 (100)	210 (100)			54 (100)	32 (100)		
	Allele								
	C	510 (80.7)	339 (80.71)			96 (88.89)	56 (87.5)		
	T	122 (19.3)	81 (19.29)			12 (11.11)	8 (12.5)		

MMP, matrix metalloproteinase; *p* value, significance level (alpha = 0.05); *p* value HWE, significance level (alpha = 0.05) by Hardy-Weinberg equilibrium.

^1^ *p* = 0.018.

^2^ *p* = 0.01.

^3^ *p* = 0.023.

**Table 4:** Binomial logistic regression analysis in the patients with pituitary adenoma (PA) and in the control group.

Model	Genotype	OR (CI 95%)	*p* value	AIC
Codominant	C/C	1		489.539
	C/T	0.345 (0.182–0.654)	**0.01**	
	T/T	1.143 (0.373–3.5)	0.815	

Dominant	C/C	1		490.616
	C/T + T/T	0.418 (0.236–0.740)	**0.003**	

Recessive	C/C + C/T	1		50.423
	T/T	1.461 (0.479–4.449)	0.505	

Overdominant	C/C + T/T	1		478.593
	C/T	0.343 (0.181–0.648)	**0.001**	

Additive	—	0.551 (0.337–0.901)	**0.018**	494.457

**Table 5:** Binomial logistic regression analysis in pituitary adenoma (PA) and the control by gender.

Model	Genotype	OR (CI 95%)	*p* value	AIC
*Males*				

Codominant	C/C	1		189.034
	C/T	0.312 (0.105–0.931)	**0.037**	
	T/T	1.495 (0.294–7.601)	0.628	

Dominant	C/C	1		189.340
	C/T + T/T	0.424 (0.167–1.077)	0.071	

Recessive	C/C + C/T	1		190.485
	T/T	1.933 (0.384–9.745)	0.424	

Overdominant	C/C + T/T	1		187.253
	C/T	0.305 (0.103–0.904)	**0.032**	

Additive	—	0.603 (0.278–1.310)	0.201	191.239

*Females*				

Codominant	C/C	1		306.013
	C/T	0.364 (0.165–0.801)	**0.012**	
	T/T	0.927 (0.196–4.381)	0.924	

Dominant	C/C	1		305.045
	C/T + T/T	0.414 (0.201–0.854)	**0.017**	

Recessive	C/C + C/T	1		311.512
	T/T	1.177 (0.251–5.525)	0.836	

Overdominant	C/C + T/T	1		304.022
	C/T	0.365 (0.166–0.802)	**0.012**	

Additive	—	0.520 (0.275–0.983)	**0.044**	306.912

**Table 6:** Frequency of MMP-9 (–1562 C/T) genotypes in the patients with pituitary adenoma (PA) and in the control group by PA invasiveness.

Gene marker	Genotype/allele	Frequency (%)					
		Control group *n* (%) (*n* = 526)	*p* HWE	Noninvasive PA group *n* (%) (*n* = 44)	*p* HWE	Invasive PA group *n* (%) (*n* = 42)	*p* HWE
*MMP-9 (*–*1562)* *Rs3918242*	Genotype						
	C/C	**340 (64.6)** ^1,2^	0.469	**36 (81.8)** ^1^	0.507	**34 (81.0)** ^2^	<**0.001**
	C/T	**169 (32.1)** ^3^		8 (18.2)		**4 (9.5)** ^3^	
	T/T	17 (3.2)		0 (0)		4 (9.5)	
	Total	526 (100)		44 (100)		42 (100)	
	Allele						
	C	849 (80.70)		80 (90.91)		72 (85.71)	
	T	203 (19.30)		8 (9.09)		12 (14.29)	

MMP, matrix metalloproteinase; *p* value, significance level (alpha = 0.05); *p* value HWE, significance level (alpha = 0.05) by Hardy-Weinberg equilibrium.

^1^ *p* = 0.021.

^2^ *p* = 0.041.

^3^ *p* = 0.001.

**Table 7:** Frequency of MMP-9 (–1562 C/T) genotype in the patients with pituitary adenoma (PA) and in the control group by PA recurrences.

Gene marker	Genotype/allele	Frequency (%)					
		Control group *n* (%) (*n* = 526)	*p* HWE	Nonrecurrence PA group *n* (%) (*n* = 66)	*p* HWE	Recurrence PA group *n* (%) (*n* = 20)	*p* HWE
*MMP-9 (*–*1562) Rs3918242*	Genotype						
	C/C	**340 (64.6)** ^1^	0.469	**54 (81.8)** ^1^	0.103	16 (80.0)	0.007
	C/T	**169 (32.1)** ^2,3^		10 **(15.2)**^2^		**2 (10.0)** ^3^	
	T/T	17 (3.2)		2 (3.0)			
	Total	526 (100)		66 (100)		2 (10.0)	
	Allele					20 (100)	
	C	849 (80.70)		118 (89.39)		34 (85.0)	
	T	203 (19.30)		14 (10.61)		6 (15.0)	

MMP, matrix metalloproteinase; *p* value, significance level (alpha = 0.05); *p* value HWE, significance level (alpha = 0.05) by Hardy-Weinberg equilibrium.

^1^ *p* = 0.005.

^2^ *p* = 0.004.

^3^ *p* = 0.047.

**Table 8:** Frequency of MMP-9 (–1562 C/T) genotype in the patients with pituitary adenoma (PA) and in the control group by PA activity.

Gene marker	Genotype/allele	Frequency (%)					
		Control group *n* (%) (*n* = 526)	*p* HWE	Inactive PA group *n* (%) (*n* = 30)	*p* HWE	Active PA group *n* (%) (*n* = 56)	*p* HWE
*MMP-9 (*–*1562) Rs3918242*	Genotype						
	C/C	**340 (64.6)** ^1^	0.469	**30 (100)** ^1,3^	0.007	**40 (71.4)** ^3^	0.044
	C/T	**169 (32.1)** ^2^		**0 (0)** ^2,4^		**12 (21.4)** ^4^	
	T/T	17 (3.2)		0 (0)		4 (7.1)	
	Total	526 (100)		30 (100)		56 (100)	
	Allele						
	C	849 (80.70)		60 (100)		92 (82.14)	
	T	203 (19.30)		0 (0)		20 (17.86)	

MMP, matrix metalloproteinase; *p* value, significance level (alpha = 0.05); *p* value HWE, significance level (alpha = 0.05) by Hardy-Weinberg equilibrium.

^1^ *p* < 0.001.

^2^ *p* < 0.001.

^3^ *p* < 0.001.

^4^ *p* < 0.007.
