# Supplementary material for: Does MMP-9 Gene Polymorphism Play a Role in Pituitary Adenoma Development?
Source: Dis Markers. 2017 Jan 17;2017:5839528. doi: 10.1155/2017/5839528 (PMC5282418; doi:10.1155/2017/5839528)
Supplement: Supplementary file 1 — Binomial logistic regression analysis in noninvasive/invasive, inactive/active and nonrecurrence/recurrence PAs. [file 5839528.f1.docx]

**Suplementary data**

***Table 9.*** Binomial logistic regression analysis in noninvasive and invasive pituitary adenoma (PA) and in the control group.

| Model | Genotype | OR (CI 95%) | p value | AIC |
| --- | --- | --- | --- | --- |
| **Noninvasive** |  |  |  |  |
| Co-dominant | C/C  C/T  T/T | 1  0.447 (0.203-0.983)  0 (-) | **0.045**  0.998 | 308.535 |
| Dominant | C/C  C/T+T/T | 1  0.406 (0.185-0.892) | **0.025** | 308.035 |
| Recessive | C/C+C/T  T/T | 1  0 (-) | 0.998 | 311.145 |
| Over-dominant | C/C+T/T  C/T | 1  0.469 (0.214-1.032) | 0.060 | 309.878 |
| Additive | --- | 0.409 (0.193-0.866) | **0.020** | 307.187 |
| **Invasive** |  |  |  |  |
| Co-dominant | C/C  C/T  T/T | 1  0.237 (0.083-0.678)  2.353 (0.749-7.393) | **0.007**  0.143 | 302.361 |
| Dominant | C/C  C/T+T/T | 1  0.430 (0.195-0.948) | **0.036** | 298.548 |
| Recessive | C/C+C/T  T/T | 1  3.152 (1.010-9.835) | **0.048** | 300.429 |
| Over-dominant | C/C+T/T  C/T | 1  0.222 (0.078-0.633) | **0.005** | 302.203 |
| Additive | --- | 0.698 (0.372-1.310) | 0.263 | 302.240 |

***Table 10.*** Binomial logistic regression analysis in inactive and active pituitary adenoma (PA) and in the control group.

| Model | Genotype | OR (CI 95%) | p value | AIC |
| --- | --- | --- | --- | --- |
| **Inactive** |  |  |  |  |
| Co-dominant | C/C  C/T  T/T | 1  0 (-)  0 (-) | 0.995  0.998 | 214.237 |
| Dominant | C/C  C/T+T/T | 1  0 (-) | 0.995 | 212.237 |
| Recessive | C/C+C/T  T/T | 1  0 (-) | 0.998 | 235.609 |
| Over-dominant | C/C+T/T  C/T | 1  0 (-) | 0.995 | 215.046 |
| Additive | --- | 0 (-) | 0.995 | 212.237 |
| **Active** |  |  |  |  |
| Co-dominant | C/C  C/T  T/T | 1  0.604 (0.309-1.181)  2.000 (0.641-6.237) | 0.140  0.232 | 370.500 |
| Dominant | C/C  C/T+T/T | 1  0.731 (0.399-1.341) | 0.312 | 371.576 |
| Recessive | C/C+C/T  T/T | 1  2.303 (0.747-7.101) | 0.146 | 370.836 |
| Over-dominant | C/C+T/T  C/T | 1  0.576 (0.297-1.119) | 0.104 | 369.757 |
| Additive | --- | 0.909 (0.547-1.511) | 0.712 | 372.497 |

***Table 11.*** Binomial logistic regression analysis in non-recurrence and recurrence pituitary adenoma (PA) and in the control group.

| Model | Genotype | OR (CI 95%) | p value | AIC |
| --- | --- | --- | --- | --- |
| **Nonrecurrence** |  |  |  |  |
| Co-dominant | C/C  C/T  T/T | 1  0.373 (0.185-0.750)  0.741 (0.166-3.297) | **0.006**  0.694 | 410.785 |
| Dominant | C/C  C/T+T/T | 1  0.406 (0.212-0.779) | **0.007** | 409.409 |
| Recessive | C/C+C/T  T/T | 1  0.936 (0.211-4.143) | 0.930 | 417.933 |
| Over-dominant | C/C+T/T  C/T | 1  0.377 (0.188-0.758) | **0.006** | 408.952 |
| Additive | --- | 0.492 (0.276-0.878) | **0.016** | 411.211 |
| **Recurrence** |  |  |  |  |
| Co-dominant | C/C  C/T  T/T | 1  0.251 (0.057-1.106)  2.500 (0.531-11.762) | 0.068  0.246 | 171.102 |
| Dominant | C/C  C/T+T/T | 1  0.457 (0.151-1.387) | 0.167 | 175.346 |
| Recessive | C/C+C/T  T/T | 1  3.327 (0.714-15.498) | 0.126 | 173.731 |
| Over-dominant | C/C+T/T  C/T | 1  0.235 (0.054-1.023) | 0.054 | 170.208 |
| Additive | --- | 0.736 (0.303-1.784) | 0.497 | 175.041 |
